# Addition of Interleukin-21 for Expansion of T-Cells for Adoptive Immunotherapy of Murine Melanoma

**DOI:** 10.3390/ijms16048744

**Published:** 2015-04-20

**Authors:** Christine Kathryn Zoon, Wen Wan, Laura Graham, Harry D. Bear

**Affiliations:** 1 Department of Surgery, Virginia Commonwealth University Health System, Richmond, VA 23298, USA; 2 Department of Biostatistics, Virginia Commonwealth University School of Medicine, Richmond, VA 23298, USA; E-Mail: wwan@vcu.edu; 3 Division of Surgical Oncology, Virginia Commonwealth University Massey Cancer Center, Richmond, VA 23298, USA; E-Mails: lgraham2@mcvh-vcu.edu (L.G.); hdbear@vcu.edu (H.D.B.)

**Keywords:** adoptive immunotherapy, melanoma, IL-2, IL-7, IL-15, IL-21

## Abstract

We previously demonstrated that interleukin (IL)-7/15 was superior to IL-2 for expansion of T cells *in vitro* for adoptive immunotherapy. We sought to ascertain whether IL-21 would further improve yield and therapeutic efficacy of T cells in culture. Naïve T cell receptor (TcR) transgenic splenocytes or antigen-sensitized lymph node cells were harvested from PMEL-1 mice and exposed to bryostatin-1 and ionomycin (B/I) for 18 h. Cells were then cultured in IL-2, IL-21, IL-7/15 or IL-7/15/21 for six days. Harvested cells were analyzed by flow cytometry and used to treat C57Bl/6 mice injected intravenously with B16 melanoma. Lungs were harvested and metastases counted 14 days after treatment. Culturing lymphocytes in IL-7/15/21 increased expansion compared to IL-2 or IL-7/15. IL-21 and IL-7/15/21 increased CD8+ cells compared to IL-2 or IL-7/15. IL-21 preferentially expanded a CD8+CD44−CD62L+ T “naïve” population, whereas IL-7/15/21 increased CD8+CD44+CD62Lhigh central-memory T cells. T cells grown in IL-7/15/21 were more effective at reducing metastases than IL-2. The addition of IL-21 to IL-7/15 induced greater expansion of lymphocytes in culture and increased the yield of CD8+ T central-memory cells *vs.* IL-7/15 alone. This may have significant impact on future clinical trials of adoptive immunotherapy, particularly for generating adequate numbers of lymphocytes for treatment.

## 1. Introduction

Adoptive immunotherapy (AIT), the infusion of *ex vivo* expanded lymphocytes, has been extensively studied in animals and humans [1,2,3,4,5]. Although this therapy has demonstrated promising results in multiple murine tumor models, a regimen that optimizes both lymphocyte expansion as well as tumor regression for human therapy remains elusive. AIT takes advantage of *ex vivo* activation and expansion of T cells away from the suppressive *in vivo* tumor environment and allows for “re-programming” of the immune cells to optimize their functional status. It also allows for additional treatment of the host (e.g., host lymphocyte depletion) prior to the re-introduction of the selected cells, which may decrease immunosuppression, and optimize trafficking and/or proliferation of the infused cells.

We have shown that T cells from both naïve splenocytes and tumor antigen-sensitized draining lymph nodes (DLN) could be expanded with exposure to interleukins (IL)-7 and 15 after activation with bryostatin and ionomycin (B/I) to significantly greater numbers than the current standard approach using IL-2 alone [6]. These T cells were also able to cure melanoma metastases as effectively as, and sometimes better than, T cells grown in IL-2 [6]. Bryostatin-1 is a macrocyclic lactone derived from *Bulgula neritina*, a marine invertebrate. In culture, bryostatin activates protein kinase C, while ionomycin increases intracellular calcium [7,8,9]. When combined, these mimic signaling through the CD3/TcR complex and promote activation and proliferation of T cells [6,10,11,12,13,14,15,16]. B/I selectively activates CD62L- T cells, which represent the “sensitized” T cells capable of anti-tumor activity and is unique to B/I activation compared to anti-CD3 activation [7,15]. These T cells are similar to CAR T cells in the sense that all their CD8 T cells carry a transgenic T cell receptor that recognizes a single epitope present on B16 melanoma cells. We have also shown that this method of stimulation, combined with alternate cytokines, re-programs human T cells and NK cells, making them resistant to suppression by myeloid derived suppressor cells [10]. Phenotypically, whereas IL-2 expansion has been shown to lead to the induction of regulatory T cells and cause T cell activation induced cell death, IL-7/15 has been shown by this lab and others to support preferential differentiation of CD8+ T cells towards a central memory (T_CM_) phenotype [6,14,17]. This central memory phenotype has been suggested in multiple studies to be more effective at inducing tumor regression than terminally differentiated effector cells, which are more likely to be selectively expanded when T cells are grown in IL-2 [17,18]. Although using IL-2 to stimulate the proliferation of anti-tumor T cells in culture remains the standard clinical approach, we were encouraged by our results with IL-7/15 to add or substitute IL-21 to our expansion regimen. IL-21 is the most recently identified member of the family of cytokines that share the common gamma chain cytokine receptor with IL-2, IL-7 and IL-15 [19,20]. Preliminary experiments performed by other groups demonstrated that IL-21 has potent immunomodulatory effects on T cells as well as NK cells [21,22,23,24]. Both IL-15 and IL-21 have been shown in multiple experiments to enhance the *in vivo* anti-tumor effects of CD8+ T cells and in some cases to potentiate tumor regression [24,25,26,27,28,29,30]. Because of the promising results seen with IL-21 to date, we endeavored to discover whether B/I and IL-21 exposure alone or in combination with IL-7/15 would increase the expansion of naïve or antigen-sensitized T cells, and whether it would increase anti-tumor activity. In addition, the T cell phenotype stimulated by exposure to IL-21 has varied in studies over the last decade, with some demonstrating increase in T_CM_ cells while others claimed inhibition of this phenotype [19,31,32]. Therefore, we also performed flow cytometry analysis of cells expanded in different cytokines to elucidate which phenotypes were preferentially selected for after exposure to bryostatin, ionomycin and various cytokines.

## 2. Results and Discussion

### 2.1. Comparative Analysis of T Cell Expansion

In repeated experiments, expansion of cells from naïve splenocytes in the IL-7/15 and IL-7/15/21 groups was dramatically higher than for either IL-2 or IL-21. Whereas expansion in IL-2 ranged from 1- to 2.8-fold increase on day 6, cells grown in IL-7/15 expanded from 8.9- to 24.2-fold and in IL-7/15/21 cell numbers increased 9.2- to 37.2-fold. Averaged over five experiments, fold expansion was 1.9 for IL-2, 2.2 for IL-21, 15.0 for IL-7/15 and 23.8 for IL-7/15/21. Fold increases in expansion for IL-7/15 and IL-7/15/21 were significantly higher than for either IL-2 or IL-21 (all *p* < 0.0006). However, fold increase for IL-7/15 and IL-7/15/21 were not significantly different from each other (*p* = 0.51). DLN lymphocyte expansion demonstrated similar results. Over three experiments IL-7/15/21 consistently had the highest expansion of cell numbers ranging from 13.3 to 38.5-fold expansion compared with IL-7/15 (7.6- to 26.4-fold), IL-21 (0.9- to 3.3-fold) and IL-2 (3.7-fold). Again, expansion in IL-7/15 and IL-7/15/21 were significantly greater than in IL-2 or IL-21 (all *p* < 0.0039), but not significantly different from each other. However, there was a trend in favor of IL-7/15/21 expansion (*p* = 0.13). It is important to note that when cells were cultured for a total of 14 days, lymphocytes grown in IL-2 not only stopped expanding, but also rapidly began to die and therefore could not be included in expansion data, flow cytometry analysis, or treatment groups.

### 2.2. Comparison of T Cell Phenotype with Various Cytokine Exposure

**Figure 1:**
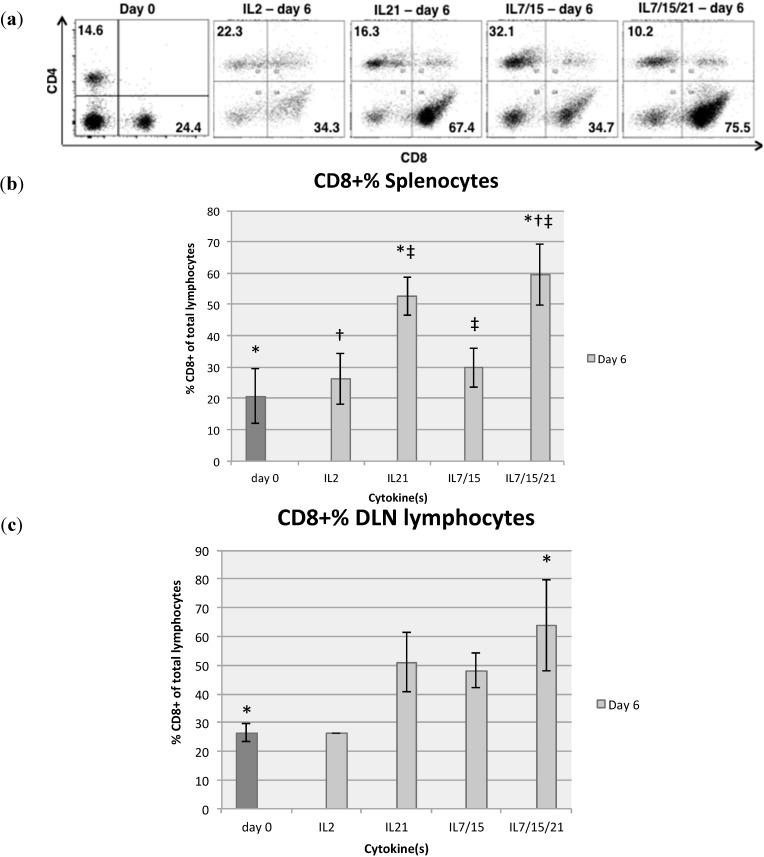
On day 0 before activation with bryostatin-1 and ionomycin (B/I) and six days after B/I activation and culture in IL-2, IL-21, IL-7/15 or IL-7/15/21, splenocytes were stained for CD4 and CD8 and analyzed by flow cytometry (**a**); Cells were FSC/SSC gated on viable lymphocytes and percentages of CD4 and CD8 (numbers in black) were determined. Average percentages of CD8+ T cells from splenocytes in five experiments, * (*vs*. day 0) = *p* < 0.01, † (*vs*. IL2) = *p* < 0.04, ‡ (*vs*. IL7/15) = *p* < 0.05 (**b**) and DLN lymphocytes in three experiments, * (*vs*. day 0) = *p* = 0.03 (**c**) expanded before and after exposure to B/I and six days of IL-2, IL-21, IL-7/15 or IL-7/15/21.

**Figure 2:**
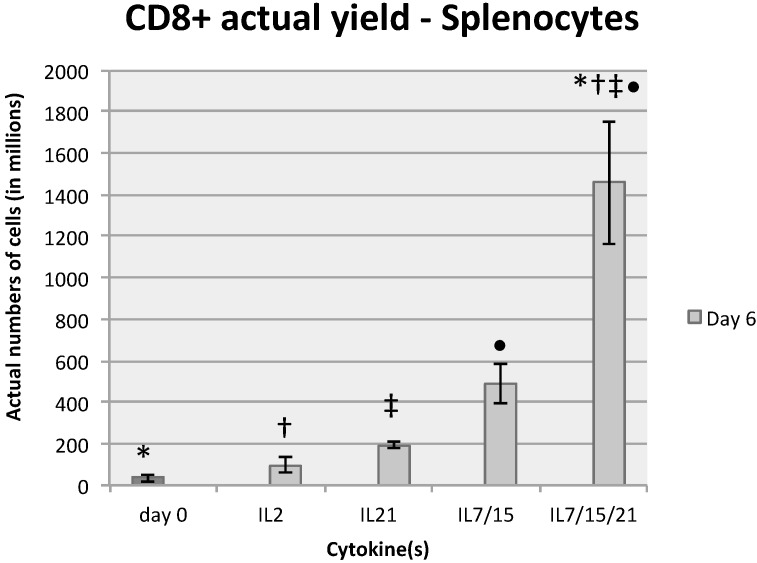
The percentages of CD8+ splenocytes were multiplied by the number of total cells expanded to obtain actual yield of cells in that subset, * (*vs*. day 0) = *p* < 0.0001, † (*vs*. IL2) = *p* < 0.0001, ‡ (*vs*. IL21) = *p* < 0.0001, • (*vs*. IL7/15) = *p* < 0.004.

At day 6 of expansion, splenocytes and DLN lymphocytes were analyzed by flow cytometry. Cells were stained for CD4, CD8, CD62L and CD44 simultaneously with appropriate nil controls as well as single color and isotype controls. In all cases, they were analyzed on the same day or within 24 h of staining and fixing. In four of four repeated experiments with splenocytes, IL-21 and IL-7/15/21 expanded the highest percentage of CD8+ T-cells on day 6 compared to IL-2 or IL-7/15 (Figure 1a,b). The average CD8+ percentages for splenocytes on day 6 of expansion were 52.7% for IL-21 and 59.6% for IL-7/15/21, compared to 20.8% on day 0 after B/I pulse (*p* < 0.01, *p* < 0.01), 26.2% for day 6 in IL-2 (*p* < 0.06, *p* < 0.04) and 29.8% for IL-7/15 (*p* < 0.05, *p* < 0.03). CD8+ lymphocyte yield from DLN was significantly higher for day 6 IL-7/15/21 compared to day 0 (*p* = 0.0304) (Figure 1c). Taking phenotypic proportions and total cell yields into account, splenocytes exposed to IL-7/15/21 produced 1456.8 million CD8+ T cells on day 6 *vs.* 487.9 million (*p* = 0.0044) for cells grown in IL-7/15, 191.9 million (*p* < 0.0001) for cells grown in IL-21, and 97.7 million (*p* < 0.0001) for cells grown in IL-2 (Figure 2). Tumor-sensitized DLN cells demonstrated a similar trend at day 6, but the differences were not as dramatic nor were they significantly different from one another: 497.3, 214.7, 53.5 and 39.5 million CD8+ cells for IL-7/15/21, IL-7/15, IL-21 and IL-2 respectively, from a starting number of 9.2 million cells on average for each group.

**Figure 3:**
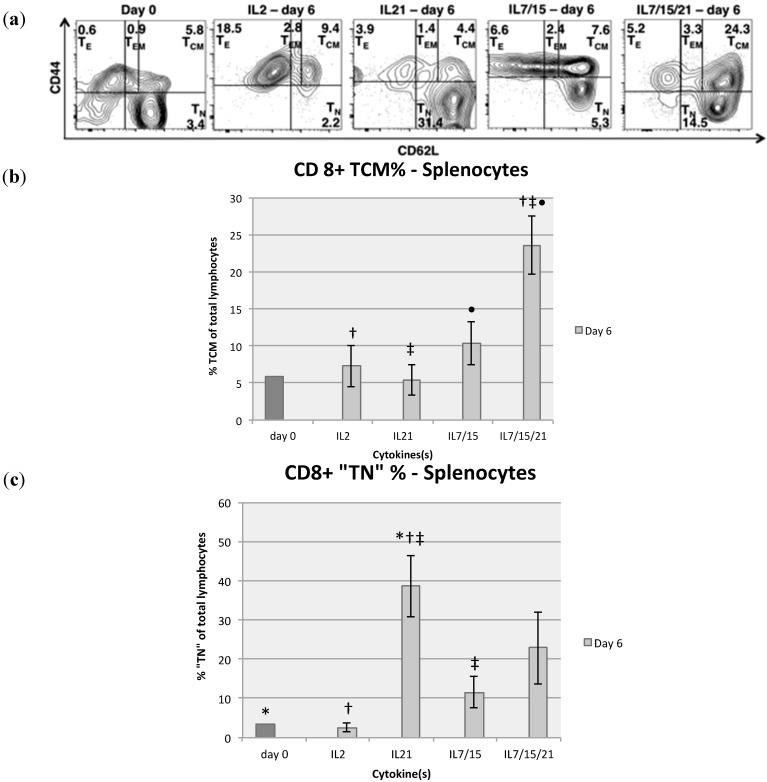
On day 0 and six days after B/I activation and culture in IL-2, IL-21, IL-7/15 or IL-7/15/21, splenocytes were stained for CD4, CD8, CD44 and CD62L and analyzed by flow cytometry to determine the percentages of T cell subsets: CD44+CD62L− T effector (T_E_), CD44+CD62Llow T effector memory (T_EM_), CD44+CD62Lhigh T central memory (T_CM_) and CD44−CD62L+ “T naïve” (“T_N_”) cells (**a**); Cells were FSC/SSC gated on viable lymphocytes and CD8+ T cells and percentages of T cell subsets (in black) were determined. Results are from a representative experiment out of five independent experiments with similar results. Average percentage of T_CM_, † (*vs*. IL2) = *p* = 0.04, ‡ (*vs*. IL21) = *p* < 0.003, • (*vs*. IL7/15) = *p* = 0.056 (**b**) or T_N_, * (*vs*. day 0) = *p* < 0.016, † (*vs*. IL2) = *p* = 0.0014, ‡ (*vs*. IL7/15) = *p* < 0.029 (**c**) for cells expanded from splenocytes in five experiments before and after B/I activation and culture for six days in IL-2, IL-21, IL-7/15 or IL-7/15/21.

**Figure 4:**
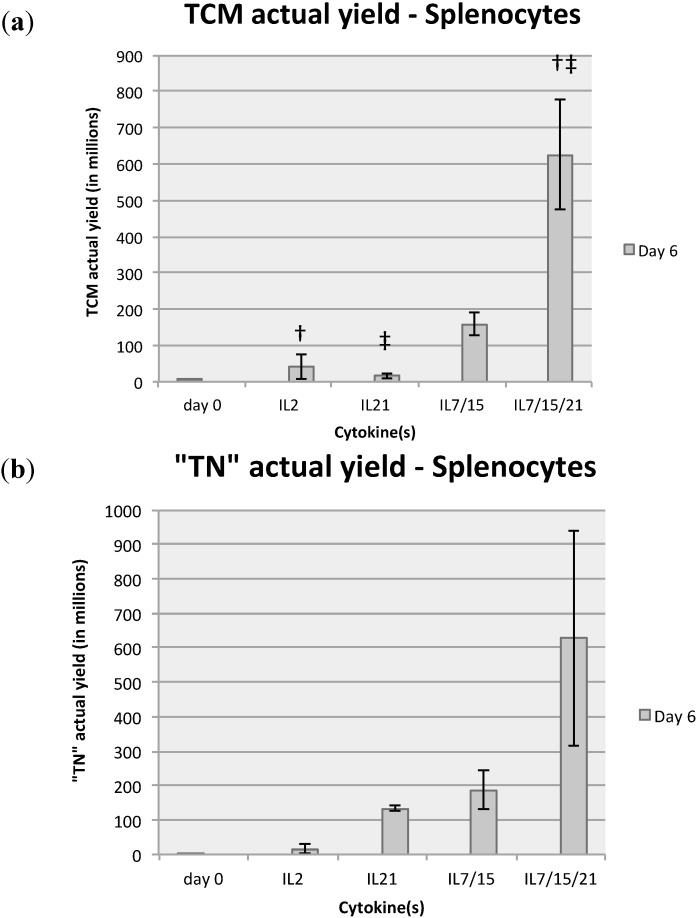
The percentages of CD8+ T_CM_, † (*vs*. IL2) = *p* < 0.01, ‡ (*vs*. IL21) = *p* = 0.004 (**a**) and CD8+ T_N_ (**b**) splenocytes were multiplied by the number of total cells expanded to obtain actual yield of cells in that subset.

Flow cytometry analysis was also performed for CD8+ T cell subsets for both splenocytes and DLN lymphocytes on day 0 and after 6 days of expansion (Figure 3a). Splenocytes grown in IL-7/15/21 consistently expanded or sustained a higher percentage of CD8+ T central memory (T_CM_) cells *vs.* all other groups (Figure 3b). Whereas the average percentage of CD8+ T_CM_ cells harvested after 6 days in IL-2 (7.3%), IL-21 (5.4%) or IL-7/15 (10.3%) were not significantly different from one another, the proportion was significantly increased with expansion in IL-7/15/21 (23.6%) compared to IL-2 (*p* = 0.0424) and IL-21 (*p* < 0.003) and trended towards significance compared to IL-7/15 (*p* = 0.056). DLN lymphocytes demonstrated a similar pattern, with IL-7/15/21 sustaining or expanding a population of CD8+ cells of which 43.1% were T_CM_ cells *vs.* 23.2% for IL-7/15, 30.5% for IL-21 and 18.1% for IL-2 at day 6 *vs.* 15.0% at day 0, but these differences were not statistically significant. An interesting finding that correlates with observations noted in previously published studies was that IL-21 alone sustained or preferentially expanded a CD8+CD62L+CD44− population of T cells, a population designated classically as “T naive” (“T_N_”) (Figure 3c) [25,31]. Splenocytes expanded for 6 days in IL-21 alone yielded 38.6% with this “T_N_” phenotype compared to IL-2 (2.6%, *p* = 0.0014), IL-7/15 (11.6%, *p* < 0.029), IL-7/15/21 (22.9%, *p* = 0.155) and day 0 (3.4%, *p* < 0.016). When the total number of cells expanded was taken into account, there were marked differences among the groups. For splenocytes, culture in IL-7/15/21 yielded an average of 625.7 million CD8+ T_CM_ cells by day 6 compared to IL-2, IL-21 and IL-7/15 [41.7 (p < 0.01), 15.8 (p = 0.0004) and 159 million cells (p = 0.125), respectively at day 6] (Figure 4a). DLN lymphocytes also responded similarly, with total CD8+ T_CM_ cells grown in IL-7/15/21 at 350 million on day 6 compared to IL-2 (27.2 million), IL-21 (31.1 million) and IL-7/15 (106.8 million) (none significant due to the limited sample size). Although the percentages of “T_N_” cells were highest with IL-21 alone, because of the greater overall expansion of T cells in the IL-7/15/21 group, that group demonstrated the highest total number of expanded “T_N_” cells, with 628 million cells at day 6 for the splenocyte group compared to 134 million cells for IL-21 (*p* = 0.09) at day 6, and 186 million cells for IL-7/15 (*p* = 0.15) (Figure 4b). DLN lymphocytes expanded with IL-7/15/21 resulted in an actual yield of 16.9 million “T_N_” cells, which was significantly higher than IL-2 expanded “T_N_” cells at 0.3 million (*p* < 0.03).

### 2.3. IFN-Gamma Release ELISA

**Figure 5:**
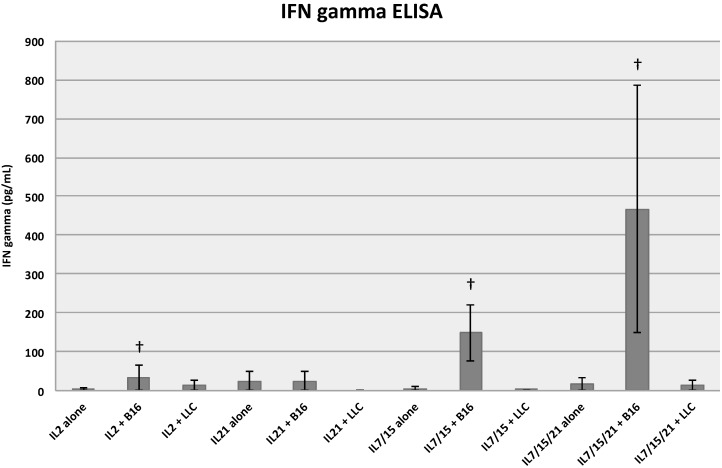
Interferon-gamma ELISA assay. B/I pulsed and IL-2, IL-21, IL-7/15 and IL-7/15/21 exposed spleen-derived T cells were co-cultured with B16 melanoma cells, Lewis lung carcinoma (LLC) cells or media alone for 24 h. The amount of IFN-γ release was significantly higher in the group exposed to IL-7/15/21 and approached significance in the group exposed to IL-7/15 than for spleen-derived T cells expanded in IL-2 or IL-21 and co-cultured with B16 cells, † (*vs*. IL2+B16) 9 = *p* < 0.04 (IL-7/15/21), *p* = 0.06 (IL-7/15).

When B/I pulsed and IL-7/15/21 exposed splenocyte T cells were co-cultured with B16 cells for 24 h, the amount of IFN-γ release was significantly higher than in those splenocyte T cells exposed to IL-2 and co-cultured with B16 cells, averaged over three experiments (*p* = 0.04) (Figure 5). T cells exposed to IL-7/15 and co-cultured with B16 cells approached significance compared to IL-2 exposed T cells cultured with B16 cells (*p* = 0.06), a finding we had demonstrated previously [6]. The amount of IFN-γ produced by IL-7/15 exposed and IL-7/15/21 exposed T cells co-cultured with B16 cells were not significantly different from each other (*p* = 0.85). Two controls were utilized, a control tumor, LLC, was used to demonstrate that the IFN-γ release from the T cells was specific to exposure to B16, and the cytokine exposed T cells alone, without tumor co-culture. Only the IL-7/15 and the IL-7/15/21 exposed T cells when co-cultured with B16 were significantly different from their LLC and alone controls (*p* < 0.05), whereas the IL-2 and IL-21 exposed T cells when co-cultured with B16 were not significantly different from their controls (*p* = 0.24–0.99).

### 2.4. Treatment of B16 Melanoma Metastases with Lymphocytes Exposed to Different Cytokines

**Figure 6:**
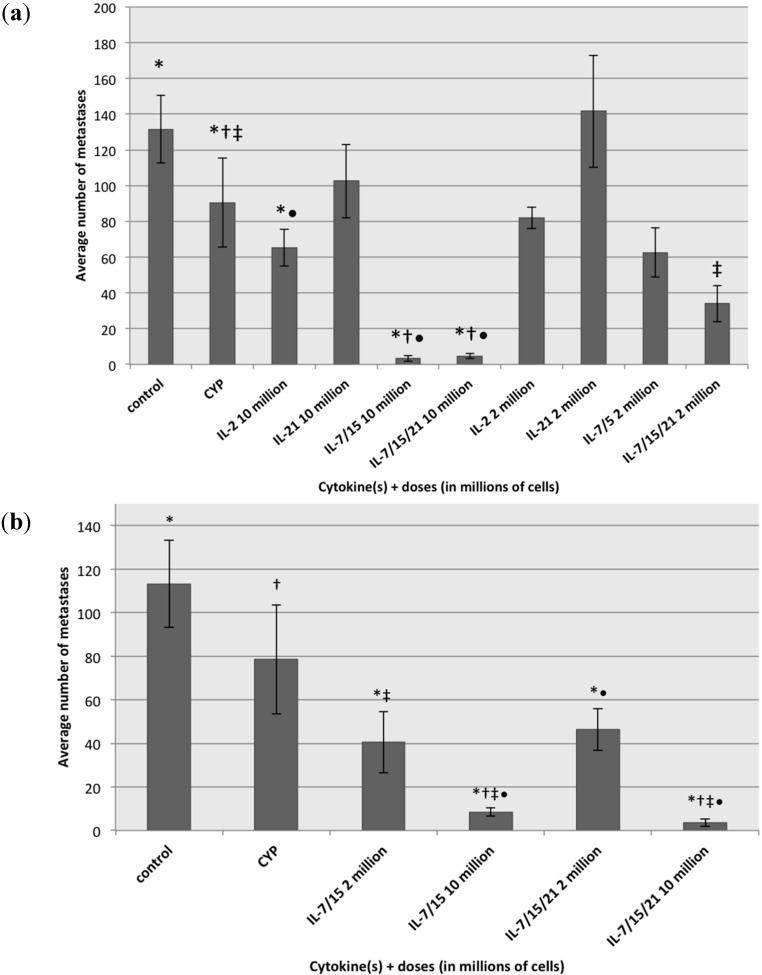
Average number of metastases in untreated mice, mice treated with cyclophosphamide (CYP) only and mice treated with cyclophosphamide and adoptive immunotherapy (AIT) with doses of either 10 million or 2 million spleen-derived T cells (**a**) activated with B/I and cultured for seven days in IL-2, IL-21, IL-7/15 or IL-7/15/21, * (*vs*. control) = *p* < 0.037, † (*vs*. CYP) = *p* < 0.0001, ‡ (CYP *vs.* IL7/15/21 2 million) = *p* = 0.002, • (*vs*. IL2 10 million) = *p* < 0.0007, and DLN derived T cells (**b**) activated with B/I and cultured for seven days in IL-7/15 or IL-7/15/21, * (*vs*. control) = *p* < 0.0019, † (*vs*. CYP) = *p* < 0.0002, ‡ (*vs*. IL7/15 2 million) = *p* < 0.02, • (*vs*. IL7/15/21 2 million) = *p* < 0.005.

Compared to the control group, mice treated with cyclophosphamide alone or cyclophosphamide plus AIT with the standard dose of spleen-derived lymphocytes (10 million cells/mouse) expanded for 7 days in IL-2, IL-7/15 or IL-7/15/21 exhibited significantly fewer metastatic lung nodules (Figure 6a) (*p* = 0.0369, *p* = 0.0330, *p* < 0.0001, *p* < 0.0001). At this dose of cells, IL-7/15 and IL-7/15/21 expanded cells were significantly better at curing metastases than cyclophosphamide alone (*p* < 0.0001, *p* < 0.0001), whereas IL-2 expanded cells were not more effective than CYP alone (*p* = 0.49). However, only the IL-7/15/21 expanded cells were significantly better than cyclophosphamide alone for curing metastases at a lower dose of T cells (2 million cells per mouse) (*p* = 0.002). In addition, when compared to AIT with 10 million cells grown in IL-2 (the current standard), IL-7/15 and IL-7/15/21 expanded cells were significantly better at curing metastases at the same cell dose (10 million) (*p* < 0.0002, *p* < 0.0007), with the majority sustaining complete cures (zero metastases seen). In our experiments, we found that AIT with cells expanded in IL-21 alone, whether at 10 million or 2 million cells, was not statistically significantly different from the control or CYP groups (*p* = 0.27, *p* = 0.59). A similar experiment was performed with DLN-derived lymphocytes (Figure 6b). Both IL-7/15 and IL-7/15/21 expanded lymphocytes were significantly better than control at the lower dose of AIT (two million cells) at curing melanoma metastases (*p* = 0.002, *p* = 0.004). However, at the standard dose of ten million cells both IL7/15 an IL7/15/21 expanded cells were capable of curing metastases significantly better than control, CYP only, and both groups at the two million cell dose (*p* < 0.02).

## 3. Discussion

These results demonstrate that culturing splenocytes and DLN lymphocytes for AIT with IL-7/15/21 results in a much higher yield of cells with equal or greater activity against melanoma metastases than those cultured in either IL-2 (the current standard cytokine for expansion) or even the combination of IL-7+15. Many studies to date have demonstrated that therapy with exogenous IL-21 or with lymphocytes exposed to IL-21 alone are superior to various combinations of cytokines [24,29,32,33,34]. Others have shown an improved effect of IL-21 combined with IL-2, IL-15 or IL-7 alone [2,22,25,31,35], However, to our knowledge this is the first study examining the effect of IL-7, -15 and -21 concurrently on *in vitro* lymphocyte expansion. Klebanoff *et al.* [1] recently reviewed which factors were associated with the most effective adoptive immunotherapy. Of the factors examined, absolute number of infused cells was strongly correlated with the magnitude of tumor regression. They also found that the least differentiated cells demonstrated the most robust anti-tumor activity. In their experience, both T central memory (T_CM_) cells and T memory stem cells (T_SCM_) led to significant delay in tumor growth compared to untreated controls or mice receiving T effector memory (T_EM_) cells. They found that T_SCM_ cells caused a more sustained reduction in tumor growth compared to T_CM_ cells. They also found that no single gamma chain cytokine, when administered exogenously, was significantly better at augmenting anti-tumor function than any other single cytokine. They go on to suggest that it is difficult to uncouple the process of cell expansion and differentiation status in that the longer the cells are expanded *ex vivo*, the more differentiated they become, and therefore, using longer durations of *in vitro* expansion to increase total cell numbers sacrifices more effective T_CM_ cells in favor of less effective terminally differentiated T_E_ cells. However, our results demonstrate that expanding splenocytes in IL-7/15/21 after activation with B/I results in a dramatic increase in the total numbers of cells, with the actual yield of CD8+ T cells capable of numbering over one billion after only 6 days of expansion from an average starting cell number of 30 million. Of these CD8+ T cells, over half were shown to be T_CM_ cells (~625 million). Splenocytes expanded in IL-7/15/21 were then subsequently found to cure metastases *in vivo* without vaccine or exogenous cytokine administration, even at low doses of cells. This finding demonstrates that even with longer times in culture and prolific expansion, lymphocytes grown in IL-7/15/21 are capable of achieving high numbers of total cells, the majority of which are T_CM_ cells that have a significant anti-tumor effect *in vivo*. In addition, this also demonstrates that the T_CM_ cells needn’t be sorted to a pure population for effective anti-tumor function. However, if desired, the total number of T_CM_ cells produced by expansion with IL-7/15/21 is such that a significant number could be enriched for therapeutic use. However, not requiring a sorted population renders adoptive immunotherapy more clinically feasible.

We consistently observed that splenocytes expanded in IL-2 or IL-21 alone did not expand well, were not effective at curing B16 metastases compared to controls, and did not produce IFN-γ when exposed to B16 tumor cells to a significantly different level compared to controls. These IL-2 and IL-21 exposed splenocytes, perhaps unsurprisingly, were the least efficacious at curing or slowing tumor growth. Overall, we found that the IL-7/15/21 cultured splenocytes had the greatest expansion, produced the greatest percentage of CD8+ T cells, the greatest number of CD8+ T_CM_ cells, had the highest amount of IFN-γ production when co-cultured with B16 cells and were most efficacious at curing metastases *in vivo*. The actual yield of CD8+ T cells and CD8+ T_CM_ cells was significantly higher in IL-7/15/21 cultured splenocytes compared to IL-7/15 splenocytes, which we had previously shown to be superior to IL-2 cultured T cells [6].

In multiple papers, it has been suggested that exposure to IL-21 alone causes lymphocytes to remain in or differentiate into a minimally differentiated phenotype (CD62L+CD44−), and that these lymphocytes have greater anti-tumor capacity relative to cells expanded in other gamma chain cytokines [25,34,36]. Gattinoni *et al.* [37] describe lymphocytes expanded in IL-21 alone, demonstrating a CD62L+CD44− phenotype described as T_SCM_-like cells. They explain that because of the unique ability of IL-21 among gamma chain cytokines to sustain STAT3 activation, this maintains a “T_SCM_-like state that is associated with high proliferative potential and long-term T cell survival” [37]. Unfortunately, in our experiments, although lymphocytes expanded in IL-21 indeed were enriched for the CD62L+CD44− phenotype, this did not translate into either higher proliferative capacity *in vitro* nor greater anti-tumor efficacy *in vivo*. This may be because our method of activation with B/I does not support development of T_SCM_-like cells after exposure to IL-21, or because T_SCM_-like cells produced by exposure to IL-21 in culture are not as effective as has been demonstrated in earlier studies.

## 4. Experimental Section

### 4.1. Mice

Virus-free C57Bl/6 mice (National Cancer Institute, National Institutes of Health, Bethesda, MD, USA) between 8 and 12 weeks of age, caged in groups of 6 or fewer, were provided with food and water ad libitum. T cell receptor (TcR) transgenic PMEL-1 mice, with T cell receptors (TcR) specific for the peptide KVPRNQDWL, from the shared melanocyte/melanoma differentiation antigen gp100, were produced from breeding pairs obtained from Jackson Laboratories (Bar Harbor, Maine). All guidelines of the Virginia Commonwealth University Institutional Animal Care and Use Committee, which conform to the American Association for Accreditation of Laboratory Animal Care and the U.S. Department of Agriculture recommendations for the care and humane experimental use of animals, were followed.

### 4.2. Tumor Cell Lines

B16-GMCSF and B16-F10 tumor cell lines were kindly provided by Richard Dutton (Trudeau Institute, Saranac Lake, NY, USA) and by Rodney Prell (Cell Genesys, Inc., South San Francisco, CA, USA), respectively. Melanoma cells were cultured in complete RPMI 1640 with 10% heat-inactivated fetal calf serum, 1 mM sodium pyruvate, 0.1 mM nonessential amino acids, 0.075% sodium bicarbonate, 2 mM l-glutamine, 100 U/mL penicillin, 100 μg/mL streptomycin, 10 mM Hepes buffer, and 5 × 10^−5^ M 2-mercaptoethanol (Sigma, St. Louis, MO, USA). All cells were incubated in 250 mL T-flasks (PGC, Gaithersburg, MD, USA) at 37 °C in humidified air with 5% CO_2_. Tumor cells were harvested from culture with 0.05% trypsin-EDTA (Invitrogen, Grand Island, NY, USA) washed twice with 1× PBS and re-suspended in 1× PBS for inoculation of mice.

### 4.3. Draining Lymph Node Sensitization

PMEL-1 mice were inoculated in one hind footpad with 5 × 10^5^ B16-GMCSF cells. Ten days after footpad vaccination, popliteal tumor draining lymph nodes (DLN) were harvested under sterile conditions.

### 4.4. Lymphocyte Activation and in Vitro Expansion

Draining lymph nodes (DLNs) or spleens were harvested and dispersed into single cell suspensions in complete RPMI media at 1 × 10^6^ cells/mL. Splenic mononuclear cells were enriched by Lympholyte-M density gradient centrifugation and residual erythrocytes were removed by suspension in ammonium chloride potassium (ACK). These cells were then activated by incubation with 5 nM bryostatin-1 (provided by the National Cancer Institute, Bethesda, MD, USA) and 1 μM ionomycin (Calbiochem, San Diego, CA, USA) (B/I) and 80 U/mL of rIL-2 (Chiron, Emeryville, VA, USA) at 37 °C for 18 h. Cells were washed three times with warm complete RPMI and re-suspended at 1–2 × 10^6^ cells/mL with either 40 U/mL of rIL-2, 5 ng/mL of IL-21 (BD Biosciences, San Jose, CA, USA), 5 ng/mL each of IL-7 (BD Biosciences, San Jose, CA, USA) and IL-15 (BD Biosciences, San Jose, CA, USA), or 5 ng/mL each of IL-7, IL-15 and IL-21. The cells were allowed to proliferate in culture for an additional 6–7 days and were split every 2–3 days in order to maintain 1× 10^6^–2 × 10^6^ cells/mL concentration and additional cytokine was added at 40 U/mL or 5 ng/mL with each split.

### 4.5. Adoptive Immunotherapy

For B16 melanoma lung metastases, mice were inoculated with 250,000 B16 melanoma cells intravenously (IV) 4 days prior to AIT. Mice were pretreated on the day prior to AIT with 100 mg/kg of intraperitoneal (IP) cyclophosphamide (CYP, Mead Johnson, Princeton, NJ, USA). AIT consisted of IV infusion of lymphocytes from cultures at either 2 or 10 million cells/mouse harvested 7 days after activation *in vitro*. No systemic cytokines or vaccines were administered to the recipient mice. In all AIT experiments, mice were euthanized by CO_2_ inhalation 14 days after tumor cell infusion and lungs were removed, fixed in 10% formaldehyde, and melanoma lung nodules were counted under a dissecting microscope, as previously described [38].

### 4.6. Flow Cytometry

T cells isolated from DLN and spleens were stained with a panel of antibodies on day 0 (immediately after B/I activation) and on day 6 after activation *in vitro*. These stained cells were analyzed by multicolor flow cytometry for surface markers on a FACSAria Canto flow cytometer. Fluorescently labeled antibodies directed against the following markers were obtained from Biolegend and eBiosciences: CD4, CD8, CD62L, and CD44. Appropriate isotype controls were used in all cases. T cell subsets analyzed were T effector (T_E_) CD44+CD62L−, T effector memory (T_EM_) CD44+CD62Llow, T central memory (T_CM_) CD44+CD62Lhigh and “T naïve” (“T_N_”) CD44–CD62L+, as described in earlier studies and reviews [39,40,41].

### 4.7. IFN-γ ELISA

Splenocytes were cultured with B/I and cytokines for 7 days as described above. The cells were then harvested, washed and cultured in complete medium at a ratio of 10:1 with irradiated B16 tumor cells or Lewis lung carcinoma (LLC) irradiated cells, as a control, for 24 h. Splenocytes alone, tumor cells alone and media alone were also cultured concurrently as controls. Supernatants were then collected and stored at −80 °C until assay. IFN-γ was detected using a Mouse IFN-γ ELISA kit (BD Pharmingen, San Jose, CA, USA) according to the manufacturer’s protocol and performed in duplicate.

### 4.8. Statistical Analysis

At least five mice were included in each experiment. Each outcome was summarized with basic descriptive statistics such as mean and standard deviation for each cytokine. Repeated measures analysis of variance through a linear mixed model was used to compare the various cytokine conditions in fold increase cell numbers on day 6 from baseline, in each T cell phenotype (including both percentage and total number of cells of CD4 and CD8, and T_E_, T_EM_, T_CM_, and T_N_), in number of lung melanoma metastases, and in ELISA. Pairwise comparisons of the various cytokines were made and tested at type I error of 5% by a *t*-test with the random measurement errors estimated by the linear mixed model. Normality was checked for each analysis. Natural logarithm was used in the data of fold increase of T cell expansion, ELISA, and percentage and total numbers of T cell phenotypes, and square root was used in number of lung melanoma metastases for normality. SAS 9.2 was used for all analyses.

## 5. Conclusions

In conclusion, we demonstrate here that lymphocytes expanded in the triple combination of IL-7/15/21 have a large proliferative capacity, significantly greater than the current standard of IL-2 expansion, and are capable of producing billions of CD8+ T cells and hundreds of millions of CD8+ T_CM_ cells from a starting population of only tens of millions of lymphocytes total. These IL-7/15/21 expanded lymphocytes are capable of inducing regression of metastatic B16 melanoma, even at low doses of cells. In addition, these effects are seen when the total expanded lymphocyte population is utilized for therapy, without the need for sorting a specific phenotypic subset of T-cells and without utilizing exogenous cytokine or vaccinations *in vivo*. These results show great promise for producing large numbers of effective anti-tumor T cells for future human trials. Also, although demonstrating similar phenotypic characteristics to T_SCM_-like cells, we found that lymphocyte expansion in IL-21alone improved neither their ability to proliferate *in vitro* nor their ability to cause tumor regression *in vivo*. It is therefore unclear whether the CD62L+CD44− IL-21 expanded lymphocytes are indeed T_SCM_-like cells.

## References

[1] Klebanoff C.A., Gattinoni L., Palmer D.C., Muranski P., Ji Y., Hinrichs C.S., Borman Z.A., Kerkar S.P., Scott C.D., Finkelstein S.E. (2011). Determinants of successful CD8+ T-cell adoptive immunotherapy for large established tumors in mice. Clin. Cancer Res..

[2] Pouw N., Treffers-Westerlaken E., Kraan J., Wittink F., ten Hagen T., Verweij J., Debets R. (2010). Combination of IL-21 and IL-15 enhances tumour-specific cytotoxicity and cytokine production of TCR-transduced primary T Cells. Cancer Immunol. Immunother..

[3] Huarte E., Fisher J., Turk M.J., Mellinger D., Foster C., Wolf B., Meehan K.R., Fadul C.E., Ernstoff M.S. (2009). *Ex vivo* expansion of tumor specific lymphocytes with IL-15 and IL-21 for adoptive immunotherapy in melanoma. Cancer Lett..

[4] Bear H.D., Roberts J., Cornell D., Tombes M.B., Kyle B. (2001). Adoptive immunotherapy of cancer with pharmacologically activated lymph node lymphocytes: A pilot clinical trial. Cancer Immunol. Immunother..

[5] Liu S., Riley J., Rosenberg S., Parkhurst M. (2006). Comparison of common γ-chain cytokines, interleukin-2, interleukin-7, and interleukin-15 for the *in vitro* generation of human tumor-reactive T lymphocytes for adoptive cell transfer therapy. J. Immunother..

[6] Le H.K., Graham L., Miller C.H., Kmieciak M., Manjili M.H., Bear H.D. (2009). Incubation of antigen-sensitized T lymphocytes activated with bryostatin 1 + ionomycin in IL-7 + IL-15 increases yield of cells capable of inducing regression of melanoma metastases compared to culture in IL-2. Cancer Immunol. Immunother..

[7] Chatila T., Silverman L., Miller R., Geha R. (1989). Mechanisms of T cell activation by the calcium ionophore ionomycin. J. Immunol..

[8] Kazanietz M.G., Lewin N.E., Gao F., Pettit G.R., Blumberg P.M. (1994). Binding of [26–3H]bryostatin 1 and analogs to calcium-dependent and calcium-independent protein kinase C isozymes. Mol. Pharmacol..

[9] Pettit G.R., Herald S.L., Doubek D.L., Arnold E., Clardy J. (1982). Isolation and structure of bryostatin 1. J. Am. Chem. Soc..

[10] Payne K.K., Zoon C.K., Wan W., Marlar K., Keim R.C., Kenari M.N., Kazim A.L., Bear H.D., Manjili M.H. (2013). Peripheral blood mononuclear cells of patients with breast cancer can be reprogrammed to enhance anti-HER-2/Neu reactivity and overcome myeloid-derived suppressor cells. Breast Cancer Res. Treat..

[11] Kmieciak M., Basu D., Payne K.K., Toor A., Yacoub A., Wang X.Y., Smith L., Bear H.D., Manjili M.H. (2011). Activated NKT cells and NK cells render T cells resistant to myeloid-derived suppressor cells and result in an effective adoptive cellular therapy against breast cancer in the FVBN202 transgenic mouse. J. Immunol..

[12] Kmieciak M., Toor A., Graham L., Bear H.D., Manjili M.H. (2011). *Ex vivo* expansion of tumor-reactive t cells by means of bryostatin 1/ionomycin and the common γ chain cytokines formulation. J. Vis. Exp..

[13] Miller C.H., Graham L., Bear H.D. (2010). Phenotype, functions and fate of adoptively transferred tumor draining lymphocytes activated *ex vivo* in mice with an aggressive weakly immunogenic mammary carcinoma. BMC Immunol..

[14] Cha E., Graham L., Manjili M.H., Bear H.D. (2010). IL-7 + IL-15 are superior to IL-2 for the *ex vivo* expansion of 4T1 mammary carcinoma-specific T cells with greater efficacy against tumors *in vivo*. Breast Cancer Res. Treat..

[15] Chin C.S., Miller C.H., Graham L., Parviz M., Zacur S., Patel B., Duong A., Bear H.D. (2004). Bryostatin 1/Ionomycin (B/I) *ex vivo* stimulation preferentially activates l-selectinlow tumor-sensitized lymphocytes. Int. Immunol..

[16] Parviz M., Chin C.S., Graham L.J., Miller C., Lee C., George K., Bear H.D. (2003). Successful adoptive immunotherapy with vaccine-sensitized T cells, despite no effect with vaccination alone in a weakly immunogenic tumor model. Cancer Immunol. Immunother..

[17] Klebanoff C.A., Gattinoni L., Torabi-Parizi P., Kerstann K., Cardones A.R., Finkelstein S.E., Palmer D.C., Antony P.A., Hwang S.T., Rosenberg S.A. (2005). Central memory self/tumor-reactive CD8+ T cells confer superior antitumor immunity compared with effector memory T cells. Proc. Natl. Acad. Sci. USA.

[18] Gattinoni L., Klebanoff C.A., Palmer D.C., Wrzesinski C., Kerstann K., Yu Z., Finkelstein S.E., Theoret M.R., Rosenberg S.A., Restifo N.P. (2005). Acquisition of full effector function *in vitro* paradoxically impairs the *in vivo* antitumor efficacy of adoptively transferred CD8+ T Cells. J. Clin. Investig..

[19] Parrish-Novak J., Dillon S.R., Nelson A., Hammond A., Sprecher C., Gross J.A., Johnston J., Madden K., Xu W., West J. (2000). Interleukin 21 and its receptor are involved in NK cell expansion and regulation of lymphocyte function. Nature.

[20] Spolski R., Leonard W.J. (2008). Interleukin-21: basic biology and implications for cancer and autoimmunity. Annu. Rev. Immunol..

[21] Leonard W.J., Spolski R. (2005). Interleukin-21: A modulator of lymphoid proliferation, apoptosis and differentiation. Nat. Rev. Immunol..

[22] Wolfl M., Merker K., Morbach H., van Gool S.W., Eyrich M., Greenberg P.D., Schlegel P.G. (2011). Primed tumor-reactive multifunctional CD62L+ human CD8+ T cells for immunotherapy. Cancer Immunol. Immunother..

[23] Singh H., Figliola M.J., Dawson M.J., Huls H., Olivares S., Switzer K., Mi T., Maiti S., Kebriaei P., Lee D.A. (2011). Reprogramming CD19-Specific T Cells with IL-21 Signaling can improve adoptive immunotherapy of B-lineage malignancies. Cancer Res..

[24] Chapuis A.G., Ragnarsson G.B., Nguyen H.N., Chaney C.N., Pufnock J.S., Schmitt T.M., Duerkopp N., Roberts I.M., Pogosov G.L., Ho W.Y. (2013). Transferred WT1-Reactive CD8+ T cells can mediate antileukemic activity and persist in post-transplant patients. Sci. Transl. Med..

[25] Hinrichs C.S., Spolski R., Paulos C.M., Gattinoni L., Kerstann K.W., Palmer D.C., Klebanoff C.A., Rosenberg S.A., Leonard W.J., Restifo N.P. (2008). IL-2 and IL-21 confer opposing differentiation programs to CD8+ T cells for adoptive immunotherapy. Blood.

[26] Brentjens R.J., Latouche J.B., Santos E., Marti F., Gong M.C., Lyddane C., King P.D., Larson S., Weiss M., Riviere I. (2003). Eradication of systemic B-cell tumors by genetically targeted human T lymphocytes co-stimulated by CD80 and Interleukin-15. Nat. Med..

[27] Di Carlo E., Comes A., Orengo A.M., Rosso O., Meazza R., Musiani P., Colombo M.P., Ferrini S. (2004). IL-21 Induces tumor rejection by specific CTL and IFN-γ-dependent CXC Chemokines in Syngeneic Mice. J. Immunol..

[28] Klebanoff C.A., Finkelstein S.E., Surman D.R., Lichtman M.K., Gattinoni L., Theoret M.R., Grewal N., Spiess P.J., Antony P.A., Palmer D.C. (2004). IL-15 enhances the *in vivo* antitumor activity of tumor-reactive CD8+ T cells. Proc. Natl. Acad. Sci. USA.

[29] Sondergaard H., Frederiksen K.S., Thygesen P., Galsgaard E.D., Skak K., Kristjansen P.E., Odum N., Kragh M. (2007). Interleukin 21 therapy increases the density of tumor infiltrating CD8+ T cells and inhibits the growth of syngeneic tumors. Cancer Immunol. Immunother..

[30] Teague R.M., Sather B.D., Sacks J.A., Huang M.Z., Dossett M.L., Morimoto J., Tan X., Sutton S.E., Cooke M.P., Ohlen C. (2006). Interleukin-15 rescues tolerant CD8+ T cells for use in adoptive immunotherapy of established tumors. Nat. Med..

[31] Zeng R., Spolski R., Finkelstein S.E., Oh S., Kovanen P.E., Hinrichs C.S., Pise-Masison C.A., Radonovich M.F., Brady J.N., Restifo N.P. (2005). Synergy of IL-21 and IL-15 in regulating CD8+ T cell expansion and function. J. Exp. Med..

[32] Kasaian M.T., Whitters M.J., Carter L.L., Lowe L.D., Jussif J.M., Deng B., Johnson K.A., Witek J.S., Senices M., Konz R.F. (2002). IL-21 limits NK Cell responses and promotes antigen-specific T cell activation: A mediator of the transition from innate to adaptive immunity. Immunity.

[33] Moroz A., Eppolito C., Li Q., Tao J., Clegg C.H., Shrikant P.A. (2004). IL-21 enhances and sustains CD8+ T cell responses to achieve durable tumor immunity: Comparative evaluation of IL-2, IL-15, and IL-21. J. Immunol..

[34] Li Y., Bleakley M., Yee C. (2005). IL-21 Influences the frequency, phenotype, and affinity of the antigen-specific CD8 T cell response. J. Immunol..

[35] Hinrichs C.S., Borman Z.A., Gattinoni L., Yu Z., Burns W.R., Huang J., Klebanoff C.A., Johnson L.A., Kerkar S.P., Yang S. (2011). Human effector CD8+ T cells derived from naive rather than memory subsets possess superior traits for adoptive immunotherapy. Blood.

[36] Albrecht J., Frey M., Teschner D., Carbol A., Theobald M., Herr W., Distler E. (2011). IL-21-treated naive CD45RA+CD8+ T cells represent a reliable source for producing leukemia-reactive cytotoxic T lymphocytes with high proliferative potential and early differentiation phenotype. Cancer Immunol. Immunother..

[37] </b>Gattinoni L., Klebanoff C.A., Restifo N.P. (2012). Paths to stemness: Building the ultimate antitumour T cell. Nat. Rev. Cancer.

[38] Lipshy K.A., Kostuchenko P.J., Hamad G.G., Bland C.E., Barrett S.K., Bear H.D. (1997). Sensitizing T-lymphocytes for adoptive immunotherapy by vaccination with wild-type or cytokine gene-transduced melanoma. Ann. Surg. Oncol..

[39] Wherry E.J., Teichgraber V., Becker T.C., Masopust D., Kaech S.M., Antia R., von Andrian U.H., Ahmed R. (2003). Lineage relationship and protective immunity of memory CD8 T cell subsets. Nat. Immunol..

[40] Parish I.A., Kaech S.M. (2009). Diversity in CD8+ T cell differentiation. Curr. Opin. Immunol..

[41] Obar J.J., Lefrancois L. (2010). Memory CD8+ T cell differentiation. Ann. N. Y. Acad. Sci..

